# Small-volume detection: platform developments for clinically-relevant applications

**DOI:** 10.1186/s12951-021-00852-1

**Published:** 2021-04-21

**Authors:** Wei-Hsuan Sung, Yu-Ting Tsao, Ching-Ju Shen, Chia-Ying Tsai, Chao-Min Cheng

**Affiliations:** 1 Chang Gung Memorial Hospital, Linkou Medical Center and Chang Gung Medical College and Chang Gung University, Taoyuan, Taiwan; 2 Department of Obstetrics and Gynecology, Kaohsiung Medical University Hospital, Kaohsiung, Taiwan; 3 Department of Ophthalmology, Fu Jen Catholic University Hospital, Fu Jen Catholic University, New Taipei City, Taiwan; 4 School of Medicine, College of Medicine, Fu Jen Catholic University, New Taipei City, Taiwan; 5 Institute of Biomedical Engineering, National Tsing Hua University, Hsinchu, Taiwan

## Abstract

Biochemical analysis of human body fluids is a frequent and fruitful strategy for disease diagnosis. Point-of-care (POC) diagnostics offers the tantalizing possibility of providing rapid diagnostic results in non-laboratory settings. Successful diagnostic testing using body fluids has been reported on in the literature; however, small-volume detection devices, which offer remarkable advantages such as portability, inexpensiveness, capacity for mass production, and tiny sample volume requirements have not been thoroughly discussed. Here, we review progress in this research field, with a focus on developments since 2015. In this review article, we provide a summary of articles that have detailed the development of small-volume detection strategies using clinical samples over the course of the last 5 years. Topics covered include small-volume detection strategies in ophthalmology, dermatology or plastic surgery, otolaryngology, and cerebrospinal fluid analysis. In ophthalmology, advances in technology could be applied to examine tear or anterior chamber (AC) fluid for glucose, lactoferrin, interferon, or VEGF. These approaches could impact detection and care for diseases including diabetic mellitus, dry-eye disease, and age-related maculopathy. Early detection and easy monitoring are critical approaches for improving overall care and outcome. In dermatology or plastic surgery, small-volume detection strategies have been applied for passive or interactive wound dressing, wound healing monitoring, and blister fluid analysis for autoimmune disease diagnosis. In otolaryngology, the analysis of nasal secretions and mucosa could be used to differentiate between allergic responses and infectious diseases. Cerebrospinal fluid analysis could be applied in neurodegenerative diseases, central neural system infection and tumor diagnosis. Other small-volume fluids that have been analyzed for diagnostic and monitoring purposes include semen and cervico-vaginal fluids. We include more details regarding each of these fluids, associated collection and detection devices, and approaches in our review.

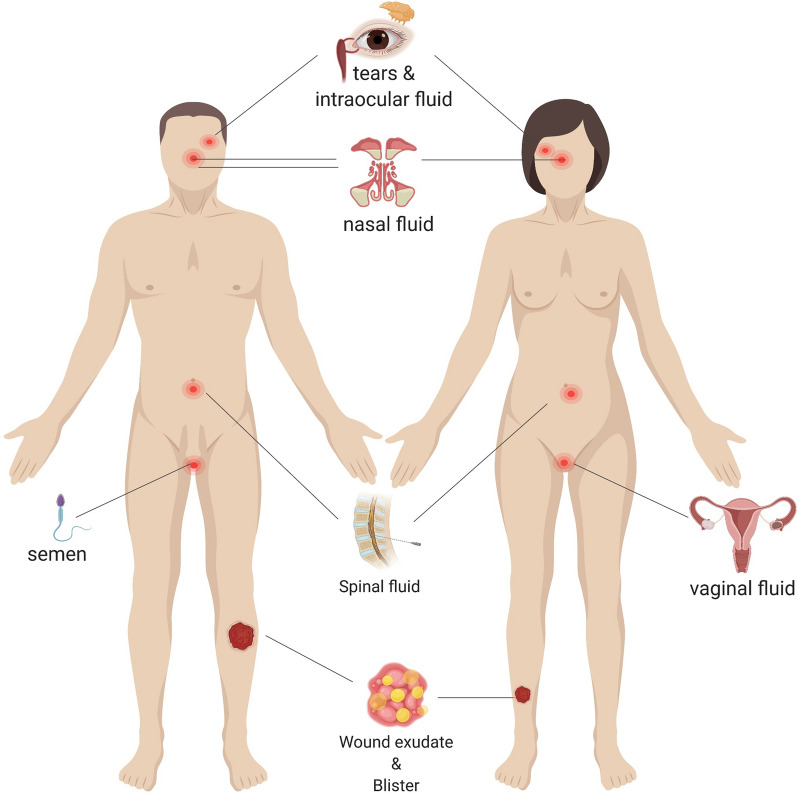

## Introduction

Biochemical analysis of human body fluid is a frequently employed strategy for effective disease diagnosis. The most common approach, widely used in hospitals and clinics, is an examination of blood chemistry. Blood sampling, whether from blood drawing or finger pricking, is relatively more invasive than tear or urine sampling [1]. Urine analysis is often an appropriate analytical strategy, especially for nephrological and urological disease diagnosis. While there are viable strategies that use large-volume samples such as blood or urine, some body fluid sources have limited volume, including those from the eye, blisters, and the cerebrospinal area, as well as body fluids such as semen and cervico-vaginal fluids. The challenges of obtaining and using samples from these areas are twofold. The first challenge is related to low volume availability, which facilitates only a limited number of tests and necessitates a suitable sampling tool as well as a sensitive and reliable testing tool. The second challenge is related to the potential health risks involved in sample collection, especially for fluids collected from the eye and the cerebrospinal area. Collecting AC fluid from the eye and aspirating cerebrospinal fluid from the interspinous space requires strict disinfection and professional personnel, and every collected sample must be carefully evaluated. Traditional, central lab analysis for disease detection requires extended time periods and large sample volumes. Finger prick blood sampling provides small volume of blood in a more convenient and less painful way. This technique has been applied to blood glucose self-monitoring in diabetic patients for many years, and is now popular worldwide. Recent studies have also described a variety of different devices that could detect infectious diseases or drug concentration from finger prick blood sampling, such as cryptococcal antigen screening [2], hepatitis C virus RNA detection [3], and infliximab concentration monitoring in inflammatory bowel disease patients [4]. These medical applications could determine infectious condition and medical concentration within a short period of time, and could provide precious information for doctors to adjust treatment immediately.

**Fig. 1:**
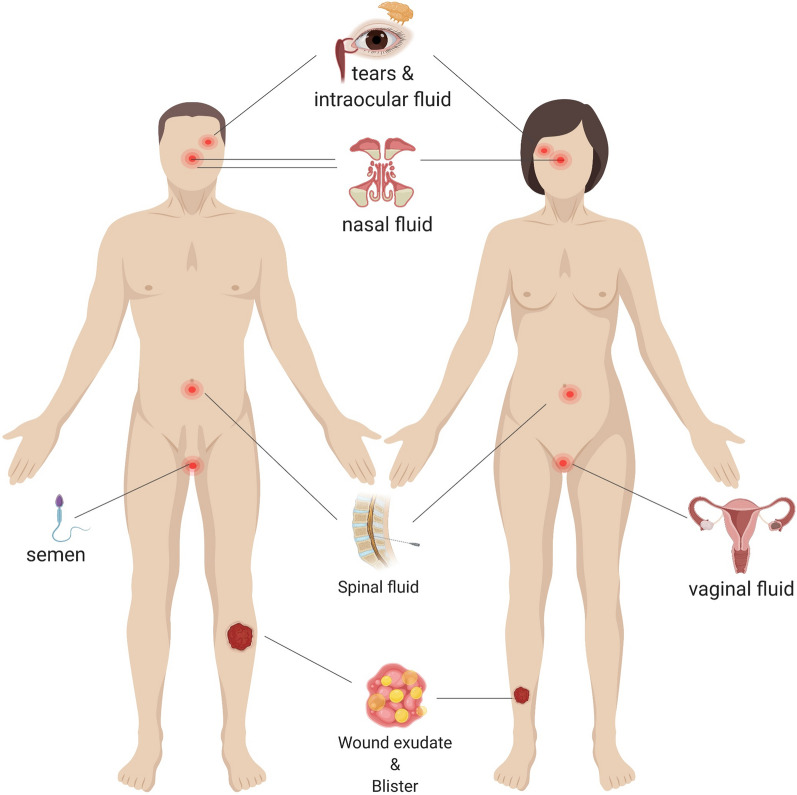
Possible sources of human fluids for fluid-based analysis. Apart from the common sources of serum and urine, several other types of body fluid can be extracted, including tears, intraocular fluid, nasal fluid, sweat, breast milk, semen, vaginal fluid, spinal fluid, and joint fluid

Advanced, point-of-care (POC) detection devices using small sample volumes have been developed to simplify disease detection and monitor disease state and treatment efficacy. The two primary benefits of such devices are: (1) decreased analysis time and clinical sample volume requirements; and (2) non-invasive, non-serological sample collection methods that reduce discomfort/pain and simplify collection. Non-serological approaches provided precedence that gave rise to the development of a variety of POC diagnostic devices [1]. There are, in fact, several collectable human body fluids that have demonstrated superior diagnostic ability for specific diseases that are hardly detected by traditional serum analysis [5, 6]. The current array of human body fluids suitable for fluid-based diagnostic analysis is provided in Fig. 1. Most of these fluids have shown great promise and impact for clinical diagnosis and follow-up analysis. Furthermore, many of them, including tear, nasal fluid, sweat, breast milk, semen, and vaginal fluid can be collected in non-invasive ways. Advances in analytical techniques and sensitive, portable platforms, have inspired greater research into non-invasive and minimally invasive methodology to meet the rising clinically relevant demand for POC diagnosis.

Portable biosensors capable of working with low-volume samples, such as those used in POC devices, not only present remarkable advantages such as portability, inexpensiveness, capacity for mass production, and small sample volume requirements, they also retain or advance diagnostic sensitivity and specificity [7] as evidenced by paper-based ELISA, a procedure that relies on a simple filter paper substrate [8]. Combining small-volume detection devices with other modern technology, e.g., smartphones, for optical and electrochemical analysis creates “all-in-one” approaches with great potential for use in laboratories, among the general population, and in remote, or resource-limited regions [9, 10]. We would, therefore, like to summarize the developments and current status of small-volume detection/diagnosis devices for clinically relevant applications.

## Applications in ophthalmology: small-volume sample analysis of tear and AC fluid

The number of microfluidic and biosensor devices suitable for ophthalmological condition assessment has increased over the last 10 years. Fluids secreted by the eye, i.e., tears and AC fluid, contain a number of detectable molecules, proteins, and antibodies. While these fluids are available in only small amounts, highly sensitive devices can be used to detect and even quantify specific fluid components as a means of diagnosing ophthalmological diseases and disease states. To date, such microfluidic approaches have been successfully used to determine glucose level, diagnose dry eye disease (DED), detect infection, and measure levels of vascular endothelium growth factor (VEGF).

### Current developments in small-volume tear sample analysis for examining diabetic mellitus (DM)

Diabetic mellitus (DM) is characterized by high blood glucose levels, and affects vessels throughout the body. In severe cases, patients experience blindness, amputation, and may require hemodialysis [11–14]. According to the World Health Organization (WHO) and other global data, an estimated 415 million people were diagnosed with DM in 2015 [15], a number that grew to 420 million in 2020 [16]. Finger-pricking is a traditional method to monitor blood glucose, but is invasive and painful. Several studies have found a correlation between glucose concentration in blood and tears [17–20], but others have found no or an indeterminate correlation [21, 22]. Despite the conflicting results, scientists still consider detection of tear glucose as a possible alternative to finger pricking. A wearable and disposable flourophor-doped contact lens [23, 24] and a contact lens embedded with an electrochemical have been used to determine tear glucose level [25]. Badugu et al. developed a glucose-sensitive silicone hydrogel contact lens to continuously monitor tear glucose level. In this glucose level sensing contact lens, the range of new boronic acid containing fluorophores (BAFs), based on the quaternized form of the quinolinium nucleus, responded to glucose within the contact lens [24]. Several review articles have noted the noninvasive merits of continuous monitoring with glucose-sensitive contact lenses [26]. A recent study showed a nanoparticle embedded contact lens (NECL) that leveraged oxidation–reduction to reflect different spectra detectable by spectrometry at different glucose levels [27]. In addition to contact lens devices, paper-based colorimetric biosensors have also been developed to detect tear glucose level. The idea for using noninvasive biosensors to determine tear glucose level was first examined by Romano et al. in 1987 [28]. Later Kim et al. demonstrated a paper-based analytical device (ìPAD) to quantify tear glucose by colorimetric detection [29]. Gabriel et al. demonstrated an ìPAD using Chitosan to detect tear glucose level in the normal population and in DM patients as a means of providing analytical reliability and accuracy [30]. In 2018, Kownaka et al. developed a NovioSense glucose sensor to continuously detect glucose level in tears. This devise used electronic components and utilized glucose oxidase as enzymatic element [31]. Tear glucose level is affected by different collection methods. Mechanical stimulation requires compression of the conjunctiva. Tear fluid may be contaminated with intercellular fluid leakage from damaged conjunctival cells, resulting in higher fluid glucose concentration. In the chemical stimulation approach, a lachrymator such as an onion is used, but this process may result in over-dilution of tear glucose. Non-stimulation approaches are the safest and most reliable methods. This process uses capillary tubes to collect 1–10 ìL tear without touching the ocular surface, but is more challenging to execute than the other two approaches described above [20, 32].

### Current developments in small-volume tear sample analysis for examining dry eye disease (DED)

Tears may also be used to measure electrolytes, enzymes, and protein. The presence and type of dry eye disease (DED), a common ocular disease affecting a number of people worldwide [33], can be evaluated by examining the level of lactoferrin, a glycoprotein in tear fluid. Autoimmune-related diseases, graft-versus-host disease (GVHD), and some medications, may lead to DED. Further, lack of lactoferrin is associated with increased overall tear concentration and corneal epithelial disorders [34]. In one experiment, terbium (Tb^3+^) was complexed to lactoferrin and subsequently used to determine lactoferrin level via fluorescence detection using a digital camera [35]. This antibody-free method had only 6% error compared to ELISA, and required only 15 min, and a 2.5 microleter sample collected by polyethylene pipette, thus providing for convenient, inexpensive, and time-saving quantification of tear lactoferrin [35]. To prevent potential error derived from different light sources and cameras, Yamada et al. developed a distance-based scheme for lactoferrin quantification using an ìPAD [36]. This distance-based approach has been used to study tear components, which were collected by polyethylene pipette and stored in Eppendorf tubes under − 30 °C [36]. Sonobe et al. investigated the correlation between tear lactoferrin and DED severity using both an iPAD and ELISA. In their study of tear fluid collected by microcapillary tubes from 24 patients, they found that tear lactoferrin was positively correlated to DED severity, and the patients with GVHD had lower levels of tear lactoferrin than non-GVHD ones [37]. The limitations of this device includes the inability to detect the extremely low lacotoferrin level in severe c-GVHD-related DED, and it lacks clarification regarding tear collection methods [37]. Other approaches useful for detecting sub-types of DED, include determining anion (Na^+^, K^+^, Ca^2+^) concentration and tear osmolality [38].

### Current developments in small-volume sample analysis for examining other ocular diseases

In recent years, some microfluidics techniques have been designed using contact lens technology to provide diagnostic or theranostic effects [39, 40]. Guan et al. designed a contact-lens-on-a-chip as a diagnostic tool for personalized medicine. This contact-lens-on-a-chip provides a way to quantify protein and microbial bioburden using only a tiny amount of tear fluid [39]. Mak et al. developed a noninvasive, theranostic contact lens for the modulation and detection of herpes simplex virus (HSV) infection. This contact lens captures and concentrates interleukin-1a (IL-1a) upregulated in HSV-1-infected patients [40]. In other research, patients with Zika virus infection demonstrated virus shedding from the lacrimal glands that was detectable in tear fluid [41]. Fungal infection may lead to pathogen-induced host response, such as a change in tear protein, after *Apergillus flavus* infection [42]. The presence of virus causes changes in tear protein composition that can be evaluated, via microfluidic techniques, to detect infection in a novel and innovative manner.

### Current developments in small-volume sample analysis based on AC fluid for examining diabetic macular edema (DME) and age-related macular degeneration (AMD)

In addition to tear fluid, AC fluid, or aqueous humor, can be used in microfluidic detection/diagnostic methodology. Unlike noninvasive tear collection, aqueous humor collection requires needle aspiration. This procedure comes with a higher risk of trauma and infection. No more than 200 μL of aqueous humor can be safely removed from the anterior chamber, but even small samples can be used to determine intraocular component concentration. Two major vision-threatening retinal diseases, diabetic macular edema (DME) and age-related macular degeneration (AMD), can be evaluated by examining intraocular VEGF level, which plays a critical role in the pathogenesis of macular edema and neovascularization in both DME and AMD [43]. The standard first-line treatment for DME and AMD is intravitreous injection of anti-VEGF, which ameliorates the increased VEGF concentration found in both conditions. Detection of aqueous humor VEGF level can be used to reflect disease condition and activity and thus monitor and appropriately treat disease early on, before further changes in macular structure and increased threat of vision loss [44, 45]. Paper-based ELISA (p-ELISA) has been used to detect small amounts of aqueous humor VEGF and could provide a rapid and accurate POC approach [44].

## Applications in dermatology and plastic surgery: current developments in small-volume sample analysis of exudate from skin

Skin, as the largest organ of the human body, protects the body against exogenous chemicals and acts as the first-line physical barrier against pathogenic microorganisms [46]. Skin lesions may compromise the barrier functions of the skin, providing potential portals of entry for infection. Wound infections may lead to a significant increase in attributable costs, higher mortality, longer hospitalization, and delayed wound healing [47]. Therefore, prevention and ascertainment of infection as well as determination of severity are critical for appropriate wound management and classification [48]. Continuous monitoring of the wound is the primary concern for patients dealing with non-healing wounds, which characteristically fail to complete the normal stages of healing, i.e., vascularization, granulation, and re-epithelialization [49]. Qualitative wound assessment is based upon gross clinical appearance via visual inspection, which could be subjective and inconsistent due to variations in lighting and distance [50]. Over the three healing stages, the relevant parameters for wound healing such as pH value, uric acid, Flightless I protein and C-reactive protein (CRP) concentration in wound exudates change accordingly. These parameters can be used to indicate healing stage and may identify healing difficulties [50–53]. Wound sensors have been more fully developed than detection sensors for other body fluids. This may be due to the fact that they can be applied directly to a wound for sample collecting and ample wound exudate exists. Notably, the range of wound fluid sample volume required for analyte detection, 0.5 to 50 µL, is relatively broad [54]. Approaches that employ wearable biosensors for monitoring wound exudate components continue to be investigated for potential use in medical diagnostics [55].

### Wound monitoring with passive dressings

**Fig. 2:**
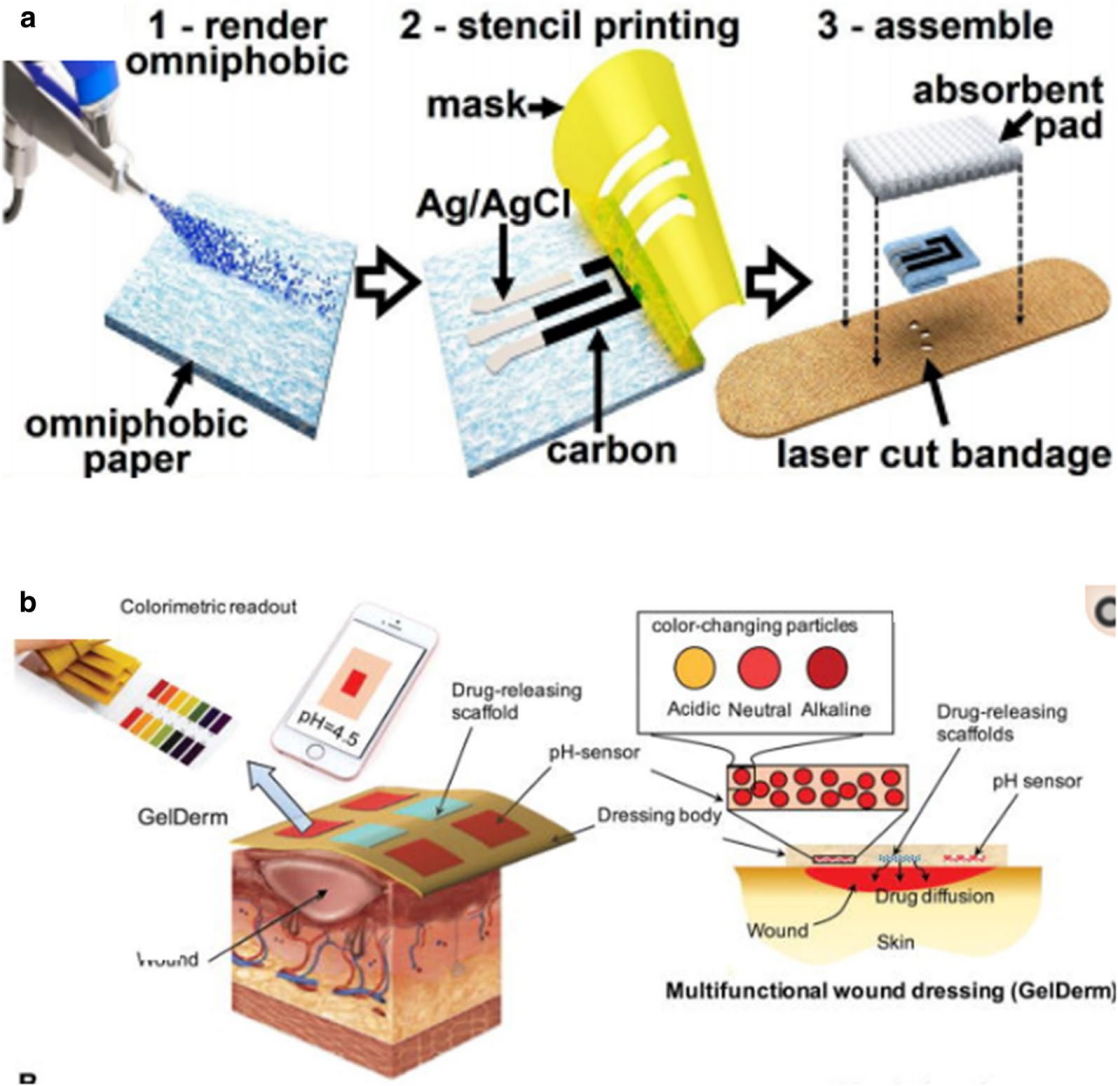
**a** Passive, smart bandage dressing fabrication and assembly process using Whatman #1 paper with a 2% solution of RF SiCl3 in IPA, stencil printing with Ag/AgCl inks and laser. **b** Interactive dressing such as hydrogel for treatment of epidermal wounds and drug-eluting components

Passive non-occlusive gauze and tulle dressings can be used to cover the wound and restore function [56]. However, such dressings are permeable toward bacteria and may adhere to the wound sometimes causing fresh trauma upon removal [56]. As for bandage-like biosensors, they are usually lightweight, flexible, breathable, easy to apply, and disposable, making them useful for both collecting and analyzing wound fluid [57]. Omniphobic paper-based smart bandages (OPSBs), for example, are designed to monitor the status of chronic wounds and to detect the formation of pressure ulcers, even before the pressure-induced tissue damage becomes visible. Moreover, they wirelessly report wound status to the user or to medical personnel [57]. Smart bandages for not only monitoring but also treatment have been presented in several studies. Mostafalu et al. integrated temperature and pH sensors into flexible bandage with a stimuli-responsive, drug-releasing system comprising a hydrogel loaded with thermo-responsive drug carriers and an electronically controlled microheater [58]. Chen et al. demonstrated a smart bandage comprising luminescent porous silicon (LuPSi) particles loaded with ciprofloxacin [59] (Fig. 2). The oxidation of LuPSi in this bandage simultaneously triggers a drug release process synchronous with fluorescent intensity change in infected wounds with higher ROS and pH.

Gauze-like and pad-like biosensor dressing system have also gained much attention and have been reported on in several recent studies. A robust electrochemical sensor-on-gauze system has been developed for quantitative measurement of uric acid that demonstrated high specificity and good linearity. This system was characterized by excellent reproducibility and consistent measurements for up to 7 h [60]. A pad-like biosensor dressing system has also been developed for simultaneous pH and glucose concentration detection. This system used a fluorescent pH indicator dye, carboxynaphtho-fluorescein, and a metabolite-sensing enzymatic system based on glucose oxidase and horseradish peroxidase for wound monitoring [61].

When it comes to designing wearable wound sensor, electrode design plays an important role. Although glass electrodes are widely used for pH detection, graphene shows promise due to its high reactivity to oxygen-bearing groups such as OH^−^ and its improved electrochemical response compared to glassy carbon electrodes, graphite, and CNTs [62]. Salvo et al. developed a pH sensor using a graphene oxide (GO) layer toward monitoring diabetic foot wound [63]. In addition to graphene, polyaniline has been used to fabricate flexible and thin nanopillar array-based pH sensors by employing soft lithography using a polymeric blend to create a flexible nanopillar backbone film [64]. Moreover, flexibility and comfort have been added to the design equation as means of bridging the gap between laboratory-tested systems and clinical applications. For example, one biosensor designed to detect vascular endothelial growth factor (VEGF) was printed with functional electrodes on flexible fibroin substrates using contact photolithography [65]. In addition to factors of comfort that improve compliance for wound biosensors, consideration must be given to material cytotoxicity as a means of evaluating true biocompatibility for continuous wound monitoring applications [66].

### Wound monitoring with interactive dressings

Interactive dressings are semi-occlusive or occlusive, available in forms such as films, foam, hydrogel, and hydrocolloid [58]. Different interactive dressings have various properties. For example, foam dressings are suitable for deep wounds, diabetic ulcers, minor burns, and venous insufficiency ulcers due to heavy wound exudate absorbance [67]. The transparency of the film allows clinicians to visualize the wound healing process. Hydrogel dressings maintain the moisture level in the wound environment by using a swelled-water hydrophilic polymer [68]. The anaerobic environment provided by hydrocolloid dressings has been shown to improve the hypertrophic graduation of tissue [69]. From these findings, it is clear that selection of proper interactive dressings in combination with biosensors capable of monitoring physiological information of wounds are technologically possible and can improve wound healing.

Hydrogels offer an advantage over other wound dressing materials for wound monitoring because they easily entrap and minimally affect active molecules, they assist in wound moisture maintenance, their transparency facilitates wound observation, and they don’t have to be removed during wound closure [58]. Hydrogels have consequently been intensively studied as carriers of sensing modalities for wound status screening [70]. Recently, a pH-responsive multifunctional hydrogel dressing (GelDerm) capable of releasing antibiotic agents at the wound site has been suggested for monitoring and managing trauma-, surgery- and diabetes-related chronic and acute injuries [71]. Another cleverly designed hydrogel-based wound dressing leveraged the pH-responsive fluorescence resonance energy transfer transition of Cyanine3 (Cy3) and Cyanine5 (Cy5) to monitor bacterial infection and provide on-demand treatment in the presence of infection via near infrared (NIR) light-triggered antibiotic release [72]. Poor tissue oxygenation is linked to wound healing. As a result, Wisniewski et al. developed tissue-integrating oxygen sensors for tracking oxygen in real-time with injectable, tissue-integrating, biocompatible, and small hydrogel-based microsensors that have been shown to overcome the foreign body response for long-term sensing [73].

### Current developments in small-volume sample analysis of blister fluid for examining autoimmune blistering skin disease

Bullous pemphigoid (BP) is one of the most common bullous autoimmune diseases. Laboratory diagnosis of BP relies on direct immunofluorescence (DIF) examination of linear antibody or complement deposition at the basement-membrane zone of a skin biopsy sample, as well as serologic tests with indirect immunofluorescence (IIF) studies and ELISA for the detection of circulating antibodies in serum. Advances in biomaterials and biomedical devices are making great strides toward developing less complex and comparably suitable methods for BP testing. Paper-based ELISA, for instance, has been used to detect the NC16A autoimmune antibody, with only 2 µL of blister fluid [74]. Further, as an alternative to serology performed on blood serum, serology may be performed on blister fluid. Biochip-based tests using blister fluid to detect antibodies, such as BP180, are appropriate initial approaches to the diagnostic workup of patients with suspected BP, especially for fragile elderly patients with poor venous access [75].

## Applications in otolaryngology: small-volume sample analysis of nasal secretions and nasal mucosa

Nasal mucosa and nasal secretions are the first line of defense for the respiratory tract and are responsible for airborne pollutant clearance and microbial prevention. Therefore, analysis of the abundant immunological markers, cytokines, and proteins in the nasal mucosa and nasal secretions may provide clinical value for diagnosis and monitoring of respiratory tract diseases [76]. Furthermore, nasal secretions, which can provide direct information about local inflammatory activity, have been widely used in studies of nasal and sinus diseases, especially allergic rhinitis [77–79] and upper airway infections [80, 81]. However, advances in the development of nasal secretion biochemical analysis has stagnated as a result of the high variance in nasal secretions between individuals, as well as individually unstable, daily changes [82] and unpredictable dilution factors associated with traditional collection methods. A novel nasal secretion collection method without dilution accompanied with precise small-volume sample analysis has yet to be developed.

### Current developments in small-volume sample analysis of nasal secretions for examining allergic and infectious disease

**Fig. 3:**
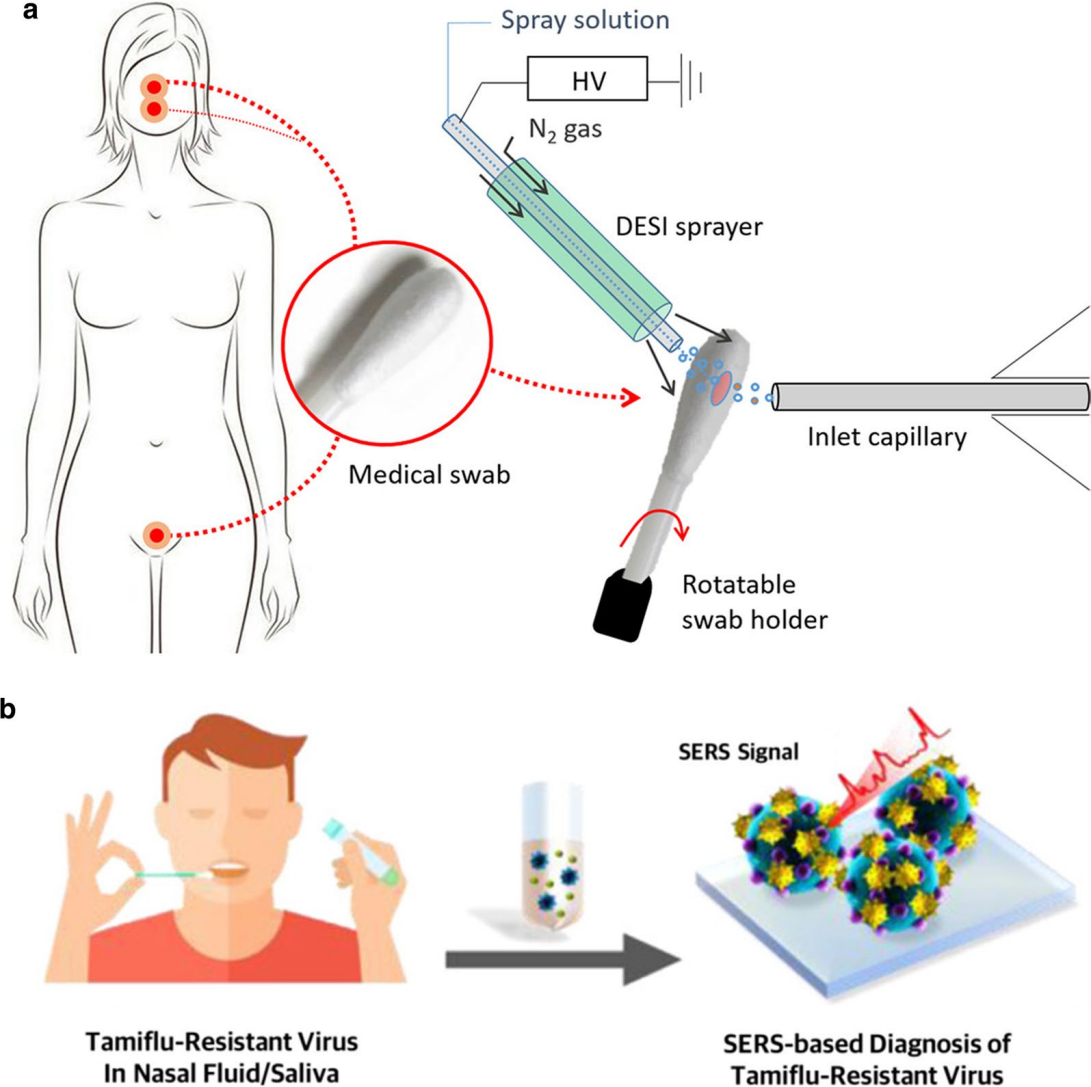
The development of small-volume sample analysis techniques for nasal fluid analysis. **a** Advanced desorption electrospray ionization mass spectrometry (DESI-MS) analysis combined with current nasal fluid absorption methodology to provide direct mucosal diagnosis based on small-volume, unprocessed mucosal sample. Charged droplets for mucosal sample extraction and transportation to a mass spectrometer [76]. **b** Advanced surface-enhanced Raman scattering (SERS) technique combined with current nasal fluid absorption methodology to detect for oseltamivir-resistant pandemic H1N1 (pH1N1) virus in nasal fluid [84]

Among the diverse range of nasal secretion collection methods, absorption seems to be the best approach for providing sufficient undiluted sample for the detection of low-concentration immunoglobulins and inflammatory mediators [83]. Current nasal fluid analysis studies have employed existing absorption methods to develop small-volume sample analysis or developed their own undiluted sample collecting methods (Fig. 3). Jochems developed an adsorptive matrix strip and another nasal curettage method to noninvasively collect nasal lining fluid and cells without dilution [84]. These methods showed superior ability to analyze immune cells and detect cytokines compared to traditional sample collection methods. Additionally, Pruski introduced a desorption electrospray ionization mass spectrometry (DESI-MS) method as a potential POC diagnostic device approach for rapid mucosal analysis [85]. Other researchers tended to combine simple sampling methods with advanced multiplex analysis of cytokines in nasal discharge to assist the diagnosis and differential diagnosis of respiratory-related allergic or infectious disease [76–89]. Because the amount nasal mucosa acquired from general nasal swab use is very limited (a few microliters) and sticky, diluting the sample with buffer is required. Unfortunately this drastically reduces target substance concentration. For this reason, polymerase chain reaction (PCR) is typically used because it increases the amount of target analyte to provide ultra-high sensitivity. This approach has been a clinical mainstay for low-volume sample testing. However, it requires the use of sophisticated laboratory equipment and a long analytical time that limit its practicality for rapid testing and for testing in large numbers. The emergence of the COVID-19 pandemic has underscored the urgent need for a more rapid and convenient POC platform for nasal secretion testing. The solution may be found in several recent, state-of-the-art studies showing that POC platforms could be developed using a variety of alternative methodologies including nucleic acid amplification tests (NAAT), rapid antigen detection tests (RADT), and fluorescent immunoassay analyzers (FIA) [90]. Sun developed a smartphone-based microfluidic chip that used an NAAT to return live virus from nasal sampling in 30 min [91]. Abdulrahman et al. and Young et al. have found that combining a paper-based lateral flow platform with RADT or IFA could yield rapid and cost-effective results that showed good consistency with the traditional PCR results (> 90%) [92, 93].

In addition, electrochemical biosensor would be another powerful POC platform for rapid detection of SARS-CoV-2 that would be worthy of large-scale investigative studies [94]. As a novel approach for respiratory tract infectious disease diagnosis, Yam combined the easy-to-manipulate nasal swab sample collection method and a commercially available LightCycler MRSA (methicillin-resistant staphylococcus) test to provide rapid detection of nasal MRSA colonization with high sensitivity and specificity [95]. This study showed the potential of nasal secretion microanalysis for screening and controlling specific infections for public health strategies. Chotiprasitsakul further verified that the MRSA nasal swab test was helpful in infection control practices [96]. Eom used the surface-enhanced Raman scattering technique to detect the Tamiflu-resistant influenza virus in patients who got the flu [97]. This study was the first to demonstrate the diagnosis of the antiviral drug-resistant virus based on only a nasal secretion. Nasal fluid-based noninvasive screening techniques have the potential to prevent the transmission of drug-resistant bacteria and viruses.

Meng recently showed that detection of sIgE levels in nasal secretion could provide a quick diagnostic result capable of replacing blood testing as an effective and noninvasive way to assist the diagnosis of some allergic diseases while most other immunoglobulins or cytokines are not highly specific for targeted disease detection [98].

### Expanded use of small-volume sample analysis for examination of nasal secretions

In recent years, microanalysis of nasal secretions has expanded in use to not only examine local allergic disease and upper respiratory tract infection, but also to provide impact in preventative medicine, otorhinolaryngology, environmental quality evaluation, and forensic investigations.

Hirvonen provided a great example of using nasal secretion analysis to evaluate environmental exposure to molds [99]. As a primary contact surface area for airborne elements, nasal mucus easily traps and retains foreign particles. It has been used as a tool to detect a variety of things including the gunshot residues of suspected shooters in forensic investigations [100, 101]. Nasal mucus can also trap inhaled medicine. Recent studies have shown the advantages of nasal mucus analysis for cocaine abuse confirmation. It provides for noninvasive analysis and precise detection of the prototype compound instead of its metabolites [102, 103]. Even though there is no strong correlation between nasal secretions and blood, nasal fluid analysis can provide a noninvasive, biochemically analytical tool for a variety of breathing-related disease and investigations.

## Small-volume sample analysis of cerebrospinal fluid (CSF)

Cerebrospinal fluid (CSF) is the most useful biological specimen for diagnosing central nervous system (CNS) diseases, including neurodegenerative and infectious diseases. However, CSF samples are collected by an optimized, but invasive procedure, lumbar puncture [104, 105]. Further, given that most of the neurodegenerative diseases are preventable in the preclinical stage but irreversible once the symptoms appear [6], several recent studies concentrated on early stage diagnosis of CNS disease based on the biochemical analysis of CSF.

### Neurodegenerative diseases

Mohammad et al. successfully improved the diagnostic sensitivity of a test for Parkinson’s disease (PD) by using protein misfolding cyclic amplification (PMCA) technology to amplify the signal of α-synuclein (αSyn), reaching detection limits of 0.1 pg/mL for αSyn oligomers. This work demonstrated a useful biochemical tool to preclinically identify patients who may develop PD [106]. Another inspiring work is that Chao et al., who developed a label-free optical nanosensor to simultaneously detect beta-amyloid (Aβ42) and total tau (T-tau). Their chip comprised four sensors, and the optical signals were produced by the functional surface coated with 10 nm Au and either Aβ42 or T-tau antibody. As a novel POC testing device with detection limits of 7.8 pg/mL of Aβ42 and 15.6 pg/mL of T-au, it can provide early diagnosis toward Alzheimer’s disease [107].

### CNS infectious disease

CNS infectious diseases have fulminant symptoms and high prevalence in developing countries. Siyuan et al. introduced next-generation sequencing techniques to examine CSF to assist in the diagnosis of neurobrucellosis [108] and Anna et al. demonstrated real-time multiplex PCR of CSF to assist in the differential diagnosis of meningitis or encephalitis [109]. Regarding the development of CSF POC testing devices, Emily LH et al. developed a treponemal immunochromatographic strip test to assist the diagnosis of neurosyphilis [110], Geoffroy et al. introduced the use of a glucometer, Bertrand et al. introduced the Multistix 10 SG strip and iSTAT to provide rapid diagnosis of bacterial meningitis, [111, 112] and Robin exploited lens-free microscopy to assist the diagnosis of meningitis [113]. All of these POC testing devices demonstrated the advantages of being rapid, easy to handle, and diagnostic accuracy equal to that of existing gold standard methods. The development of advanced or innovative diagnostic tools may play a critical role in guiding treatment of infectious diseases and reduce the misuse of antibiotics in CNS infections.

### CNS tumor

In addition, noninvasive POC analytical approaches have been developed in recent years for examining CSF to diagnose CNS tumors—these approaches would replace invasive CNS biopsy methods [114]. The development of POC diagnostic devices for CSF analysis remains very promising.

## Small-volume sample analysis of other body fluids

### Semen analysis

**Fig. 4:**
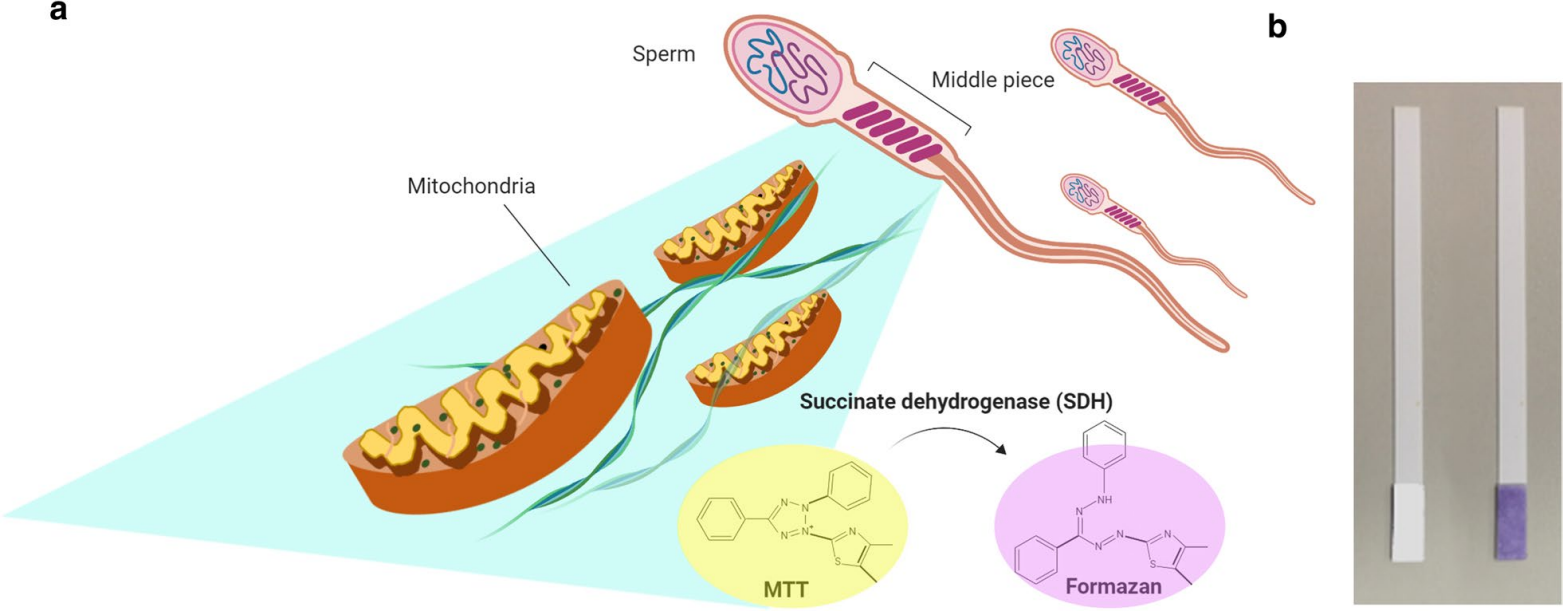
Paper-based MTT assay schematic for sperm testing. **a** The mechanism of paper-based MTT assay for sperm testing. There is an abundance of mitochondria in the middle portion of sperm. Succinate dehydrogenase (SDH) within the mitochondria can transform yellow-colored MTT into purple-colored formazon. **b** The colorimetric results of the strip, which can be interpreted by visual observation or by smartphone-based recording and analytical system. *MTT* 3-(4,5-Dimethyl-2-thiazolyl)-2,5-diphenyl-2*H*-tetrazolium bromide [120]

Semen analysis is primarily used in the field of male infertility and the median semen volume per ejaculation is about 3–4 mL. The clinical standard methods are manual microscope-based counting and computer-assisted sperm analysis (CASA) [115]. In recent years, studies have focused on developing POC diagnostic devices to overcome the psychological resistance of men who would otherwise have to go to a hospital for infertility testing. Su developed a lightweight automated semen analysis platform combining lensfree, on-chip microscopy to conduct semen analysis, which was the precedent for developing POC devices in the field [116]. Based on these technologies, automated smartphone-based semen analytical assays have been developed. Kanakasabapathy has designed a portable smartphone-based POC diagnostic device that could distribute the unprocessed semen sample on the microchip by a capillary-based tip and a rubber bulb to create negative pressure. This allowed them to image and analyse small semen sample volumes (< 35 µL) within 5 min [117]. Furthermore, a biochemical-based POC semen analytical device that combined a microfluidic paper plate with a colorimetric reaction has been developed: Matsuura developed paper-based assays for sperm counting and sperm motility assessment [118, 119]. Tsao developed a paper-based MTT assay to differentiate normal and low total motile sperm concentration for semen screening by self-assessment [120]. Using a different approach than traditional image-based analytical techniques, these biochemical-based POC diagnostic devices all loaded the reaction reagent onto the paper platform first and then directly inserted the test strips into the semen sample, which largely reduced the duration and cost (0.1 USD per test) for semen analysis (Fig. 4). This study also introduced the combination of universal image readout devices (smartphone or camera) and imaging processing software to streamLine the process. The integration of microfluidic platforms, simple image readout devices, and the internet may pave the path for future advances in semen analysis.

### Cervico-vaginal fluid

Cervico-vaginal fluid (CVF) is considered a potential source of noninvasive biomarkers for various female-specific diseases. It is characterized by its small volume, averaging only about 0.5 mL [121], and its capacity for significant influence by the microbial community [122]. Recent studies have found many cytokines and proteins that are correlated with the premature rupture of membranes including prolactin, IL-6, IL-10, TNF-α, GM-CSF, MCP1, neutrophil elastase, acetate, VDBP, TIMP-1, DKK3, RANTES, etc. [123–128]. Among these measured cytokines, IL-6 was most studied and showed an equally predictive value for intra-amniotic infection to amniotic fluid WBC count in some studies [129–133]. Musilova has used a paper-based lateral flow platform to detect vaginal fluid IL-6. The non-centrifuged vaginal fluid was directed loaded onto the platform and approximately 100 µL was adequate to conduct the test with good specificity and negative predictive value [134].

In addition premature membrane rupture, CVF cytokines can also be used to screen for lower genital tract infections, [135, 136] and cancer [137]. Appidi et al. developed a POC colorimetric assay to detect the cervical cancer. Cervico-vaginal swabs was used for sample collection and the samples were diluted in buffer to expend sample volume. After mixing the sample with HAuCl_4_ and ascorbic acid in the plastic tube, colorimetric result developed within 1 min. This is a successful study that introduced label-free gold nanoparticle based POCT in CVF [138].

However, further development of POC CVF analytical devices is hampered by the relatively low sensitivity and specificity of independent cytokines and proteins. Integrating the abundant research results and picking out a cluster of parameters for accurate diagnosis of specific lower genital tract diseases is necessary for the future development of POC CVF analysis.

### Interstitial fluid

Interstitial fluid is also informative regarding metabolites, pharmakokinetics and immunologic markers. Microneedle (MN) patch and tattoo biosensors have been used to extract and detect metabolite levels, such as glucose, cholesterol, and albumin, from the dermal interstitial fluid, which provide a minimal-invasive method to monitor diabetic mellitus and other metabolic syndrome [139, 140]. An animal study also showed that an MN patch could be used to monitor Vancomycin level and polio antibody level from dermal interstitial fluid, an approach that could be used to replace blood draw methodology [141].

## Conclusions

In laboratory diagnostics, research and developments in POC testing of small-volume samples is relatively young but offers abundant space for further progress. Studies using small-volume clinical sample detection and liquid biopsy approaches have become increasingly common and more refined. To accomplish POC detection of small volume samples, we need advanced tools. In this article, we summarized important recent advancements in POC detection technologies using human body fluids, including detection tools for AC fluid, contact lenses for examining glucose level in tears, and tools for analyzing wound and blister contents, nasal secretion, CSF, CVF, and sperm. Collection of tear fluid, nasal secretions, blister secretions, and even wound secretions from hydrogel dressings, can be executed non-invasively during routine medical care. Some procedures are invasive but would not leave permanent wounds or complications if carried out carefully. From this review, we are most encouraged and inspired to employ advanced technologies for collecting and examining AC fluids. We have published relevant related studies and our current work focuses on using AC fluid for detecting various ophthalmic diseases, such as age-related macular degeneration and dry eye. Additionally, tear fluid, which can be easily and noninvasively collected using only a paper strip, is a promising diagnostic fluid source that may become more reliable with advances in POC technology. This review provides a complete picture of the current development in this domain and further promotes the development of small-volume POC diagnostic products for both academic and commercial communities as a means of providing advances in early disease diagnosis and management.

## Data Availability

Not applicable.

## References

[1] Price CP (2001). Point of care testing. BMJ.

[2] Wake RM, Jarvis JN, Harrison TS, Govender NP (2018). Brief report: point of care cryptococcal antigen screening: pipetting finger-prick blood improves performance of immunomycologics lateral flow assay. J Acquir Immune Defic Syndr.

[3] Bielen R, Koc ÖM, Busschots D, Verrando R, Nevens F, Robaeys G (2020). Validation of hepatitis C virus RNA detection using capillary blood by finger prick (GenXpert system)-Hepatitis C fingerprick study. J Viral Hepat.

[4] Berends SE, D'Haens GRAM, Schaap T, de Vries A, Rispens T, Bloem K (2019). Dried blood samples can support monitoring of infliximab concentrations in patients with inflammatory bowel disease: a clinical validation. Br J Clin Pharmacol.

[5] Allen J, Chacko J, Donahue B, Dhall G, Kretschmar C, Jakacki R (2012). Diagnostic sensitivity of serum and lumbar CSF bHCG in newly diagnosed CNS germinoma. Pediatr Blood Cancer.

[6] Garweg JG, Jacquier P, Boehnke M (2000). Early aqueous humor analysis in patients with human ocular toxoplasmosis. J Clin Microbiol.

[7] Yen TH, Chen KH, Hsu MY, Fan ST, Huang YF, Chang CL (2015). Evaluating organophosphate poisoning in human serum with paper. Talanta.

[8] Cheng CM, Martinez AW, Gong J, Mace CR, Phillips ST, Carrilho E (2010). Paper-based ELISA. Angew Chem Int Ed Engl.

[9] Roda A, Michelini E, Zangheri M, Fusco MD, Calabria D, Simoni P (2016). Smartphone-based biosensors: a critical review and perspectives. TrAC.

[10] Calabria D, Caliceti C, Zangheri M, Mirasoli M, Simoni P, Roda A (2017). Smartphone-based enzymatic biosensor for oral fluid l-lactate detection in one minute using confined multilayer paper reflectometry. Biosens Bioelectron.

[11] Koev ST, Dykstra PH, Luo X, Rubloff GW, Bentley WE, Payne GF (2010). Chitosan: an integrative biomaterial for lab-on-a-chip devices. Lab Chip.

[12] Rinaudo M (2006). Chitin and chitosan: properties and applications. Prog Polym Sci.

[13] Shukla SK, Mishra AK, Arotiba OA, Mamba BB (2013). Chitosan-based nanomaterials: a state-of-the-art review. Int J Biol Macromol.

[14] Yi H, Wu LQ, Bentley WE, Ghodssi R, Rubloff GW, Culver JN (2005). Biofabrication with chitosan. Biomacromol.

[15] Ogurtsova K, da Rocha Fernandes JD, Huang Y, Linnenkamp U, Guariguata L, Cho NH (2017). IDF Diabetes Atlas: global estimates for the prevalence of diabetes for 2015 and 2040. Diabetes Res Clin Pract.

[16] World Health Organization. Insulin and associated devices: access for everybody: WHO stakeholder workshop, 21 and 23–25 September 2020. World Health Organization; 2020.

[17] Chen R, Jin Z, Colón LA (1996). Analysis of tear fluid by CE/LIF: a noninvasive approach for glucose monitoring. J Capill Electrophor.

[18] Daum KM, Hill RM (1984). Human tears: glucose instabilities. Acta Ophthalmol.

[19] Gasset AR, Braverman LE, Fleming MC, Arky RA, Alter BR (1968). Tear glucose detection of hyperglycemia. Am J Ophthalmol.

[20] Lane JD, Krumholz DM, Sack RA, Morris C (2006). Tear glucose dynamics in diabetes mellitus. Curr Eye Res.

[21] Sen DK, Sarin GS (1980). Tear glucose levels in normal people and in diabetic patients. Br J Ophthalmol.

[22] Taormina CR, Baca JT, Asher SA, Grabowski JJ, Finegold DN (2007). Analysis of tear glucose concentration with electrospray ionization mass spectrometry. J Am Soc Mass Spectrom.

[23] Alexeev VL, Das S, Finegold DN, Asher SA (2004). Photonic crystal glucose-sensing material for noninvasive monitoring of glucose in tear fluid. Clin Chem.

[24] Badugu R, Lakowicz JR, Geddes CD (2003). A glucose sensing contact lens: a non-invasive technique for continuous physiological glucose monitoring. J Fluoresc.

[25] Chu MX, Miyajima K, Takahashi D, Arakawa T, Sano K, Sawada S (2011). Soft contact lens biosensor for in situ monitoring of tear glucose as non-invasive blood sugar assessment. Talanta.

[26] Ascaso FJ, Huerva V (2016). Noninvasive continuous monitoring of tear glucose using glucose-sensing contact lenses. Optom Vis Sci.

[27] Kim S, Jeon HJ, Park S, Lee DY, Chung E (2020). Tear glucose measurement by reflectance spectrum of a nanoparticle embedded contact lens. Sci Rep.

[28] Romano A, Rolant F (1985). A non-invasive method of blood glucose evaluation by tear glucose measurement, for the detection and control of diabetic states. Metab Pediatr Syst Ophthalmol.

[29] Kim HJ, Jeong S, Noh H (2015). Quantitative determination of tear glucose using paper based microfluidic devices. J Korean Chem Soc.

[30] Gabriel EF, Garcia PT, Cardoso TM, Lopes FM, Martins FT, Coltro WK (2016). Highly sensitive colorimetric detection of glucose and uric acid in biological fluids using chitosan-modified paper microfluidic devices. Analyst.

[31] Geelhoed-Duijvestijn P, Vegelyte D, Kownacka A, Anton N, Joosse M, Wilson C (2020). Performance of the prototype NovioSense noninvasive biosensor for tear glucose in type 1 diabetes. J Diabetes Sci Technol.

[32] Baca JT, Finegold DN, Asher SA (2007). Tear glucose analysis for the noninvasive detection and monitoring of diabetes mellitus. Ocul Surf.

[33] International Dry Eye WorkShop Study Group (2007). The epidemiology of dry eye disease: report of the Epidemiology Subcommittee of the International Dry Eye WorkShop (2007). Ocul Surf.

[34] Danjo Y, Lee M, Horimoto K, Hamano T (1994). Ocular surface damage and tear lactoferrin in dry eye syndrome. Acta Ophthalmol.

[35] Yamada K, Takaki S, Komuro N, Suzuki K, Citterio D (2014). An antibody-free microfluidic paper-based analytical device for the determination of tear fluid lactoferrin by fluorescence sensitization of Tb3+. Analyst.

[36] Yamada K, Henares TG, Suzuki K, Citterio D (2015). Distance-based tear lactoferrin assay on microfluidic paper device using interfacial interactions on surface-modified cellulose. ACS Appl Mater Interfaces.

[37] Sonobe H, Ogawa Y, Yamada K, Shimizu E, Uchino Y, Kamoi M (2019). A novel and innovative paper-based analytical device for assessing tear lactoferrin of dry eye patients. Ocul Surf.

[38] Yetisen AK, Jiang N, Tamayol A, Ruiz-Esparza GU, Zhang YS, Medina-Pando S (2017). Paper-based microfluidic system for tear electrolyte analysis. Lab Chip.

[39] Guan A, Wang Y, Phillips KS, Li Z (2016). A contact-lens-on-a-chip companion diagnostic tool for personalized medicine. Lab Chip.

[40] Mak WC, Cheung KY, Orban J, Lee CJ, Turner AP, Griffith M (2017). Theranostic contact lens for modulation and detection of viral infection Richard Newell. Procedia Technol.

[41] Miner JJ, Sene A, Richner JM, Smith AM, Santeford A, Ban N (2016). Zika virus infection in mice causes panuveitis with shedding of virus in tears. Cell Rep.

[42] Kandhavelu J, Demonte NL, Namperumalsamy VP, Prajna L, Thangavel C, Jayapal JM (2017). Aspergillus flavus induced alterations in tear protein profile reveal pathogen-induced host response to fungal infection. J Proteom.

[43] Shams N, Ianchulev T (2006). Role of vascular endothelial growth factor in ocular angiogenesis. Ophthalmol Clin North Am.

[44] Hsu MY, Chen SJ, Chen KH, Hung YC, Tsai HY, Cheng CM (2015). Monitoring VEGF levels with low-volume sampling in major vision-threatening diseases: age-related macular degeneration and diabetic retinopathy. Lab Chip.

[45] Hsu MY, Hung YC, Hwang DK, Lin SC, Lin KH, Wang CY (2016). Detection of aqueous VEGF concentrations before and after intravitreal injection of anti-VEGF antibody using low-volume sampling paper-based ELISA. Sci Rep.

[46] Mohammadi MH, Heidary Araghi B, Beydaghi V, Geraili A, Moradi F, Jafari P (2016). Skin diseases modeling using combined tissue engineering and microfluidic technologies. Adv Healthc Mater.

[47] Bowler PG, Duerden BI, Armstrong DG (2001). Wound microbiology and associated approaches to wound management. Clin Microbiol Rev.

[48] Frykberg RG, Banks J (2015). Challenges in the treatment of chronic wounds. Adv Wound Care.

[49] Yang Y, Gao W (2019). Wearable and flexible electronics for continuous molecular monitoring. Chem Soc Rev.

[50] Salvo P, Dini V, Di Francesco F, Romanelli M (2015). The role of biomedical sensors in wound healing. Wound Med.

[51] Power G, Moore Z, O'Connor T (2017). Measurement of pH, exudate composition and temperature in wound healing: a systematic review. J Wound Care.

[52] Rajeev G, Melville E, Cowin AJ, Prieto-Simon B, Voelcker NH (2020). Porous alumina membrane-based electrochemical biosensor for protein biomarker detection in chronic wounds. Front Chem.

[53] Nischwitz SP, Bernardelli de Mattos I, Hofmann E (2019). Continuous pH monitoring in wounds using a composite indicator dressing—a feasibility study. Burns.

[54] RoyChoudhury S, Umasankar Y, Jaller J, Herskovitz I, Mervis J, Darwin E (2018). Continuous monitoring of wound healing using a wearable enzymatic uric acid biosensor. Electrochem Soc.

[55] Salvo P, Dini V, Kirchhain A, Janowska A, Oranges T, Chiricozzi A (2017). Sensors and biosensors for C-reactive protein, temperature and pH, and their applications for monitoring wound healing: a review. Sensors.

[56] Dhivya S, Padma VV, Santhini E (2015). Wound dressings—a review. Biomedicine.

[57] Pal A, Goswami D, Cuellar HE, Castro B, Kuang S, Martinez RV (2018). Early detection and monitoring of chronic wounds using low-cost, omniphobic paper-based smart bandages. Biosens Bioelectron.

[58] Mostafalu P, Tamayol A, Rahimi R, Ochoa M, Khalilpour A, Kiaee G (2018). Smart bandage for monitoring and treatment of chronic wounds. Small.

[59] Chen X, Wo F, Jin Y, Tan J, Lai Y, Wu J (2017). Drug-porous silicon dual luminescent system for monitoring and inhibition of wound infection. ACS Nano.

[60] Liu X, Lillehoj PB (2017). Embroidered electrochemical sensors on gauze for rapid quantification of wound biomarkers. Biosens Bioelectron.

[61] Jankowska DA, Bannwarth MB, Schulenburg C, Faccio G, Maniura-Weber K, Rossi RM, Scherer L (2017). Simultaneous detection of pH value and glucose concentrations for wound monitoring applications. Biosens Bioelectron.

[62] Salvoa P, Melai B, Calisi N, Paoletti C, Bellagambi F, Kirchhain A (2018). Graphene-based devices for measuring pH. Actuators B Chem.

[63] Salvo P, Calisi N, Melai B, Dini V, Paoletti C, Lomonaco T (2017). Temperature- and pH-sensitive wearable materials for monitoring foot ulcers. Int J Nanomed.

[64] Sheybani R, Shukla A (2017). Highly sensitive label-free dual sensor array for rapid detection of wound bacteria. Biosens Bioelectron.

[65] Xu M, Yadavalli VK (2019). Flexible biosensors for the impedimetric detection of protein targets using silk-conductive polymer biocomposites. ACS Sens.

[66] Bhushan P, Umasankar Y, Hutcheson JD, Bhansali S (2019). Toxicity assessment of wearable wound sensor constituents on keratinocytes. Toxicol In Vitro.

[67] Dabiri G, Damstetter E, Phillips T (2016). Choosing a wound dressing based on common wound characteristics. Adv Wound Care.

[68] Kamoun EA, Kenawy ES, Chen X (2017). A review on polymeric hydrogel membranes for wound dressing applications: PVA-based hydrogel dressings. J Adv Res.

[69] Brown MS, Ashley B, Koh A (2018). Wearable technology for chronic wound monitoring: current dressings, advancements, and future prospects. Front Bioeng Biotechnol.

[70] Francesko A, Petkova P, Tzanov T (2018). Hydrogel dressings for advanced wound management. Curr Med Chem.

[71] Mirani B, Pagan E, Currie B, Siddiqui MA, Hosseinzadeh R, Mostafalu P, Zhang YS, Ghahary A, Akbari M (2017). An advanced multifunctional hydrogel-based dressing for wound monitoring and drug delivery. Adv Healthc Mater.

[72] Qiao B, Pang Q, Yuan P, Luo Y, Ma L (2020). Smart wound dressing for infection monitoring and NIR-triggered antibacterial treatment. Biomater Sci.

[73] Wisniewski NA, Nichols SP, Gamsey SJ, Pullins S, Au-Yeung KY, Klitzman B (2017). Tissue-integrating oxygen sensors: continuous tracking of tissue hypoxia. Adv Exp Med Biol.

[74] Hsu CK, Huang HY, Chen WR, Nishie W, Ujiie H, Natsuga K, Fan ST, Wang HK, Lee JY, Tsai WL, Shimizu H, Cheng CM (2014). Paper-based ELISA for the detection of autoimmune antibodies in body fluid-the case of bullous pemphigoid. Anal Chem.

[75] Sernicola A, Russo I, Saponeri A, Alaibac M (2019). Biochip detection of BP180 autoantibodies in blister fluid for the serodiagnosis of bullous pemphigoid: a pilot study. Medicine.

[76] Joo NS, Evans IA, Cho HJ, Park IH, Engelhardt JF, Wine JJ (2015). Proteomic analysis of pure human airway gland mucus reveals a large component of protective proteins. PLoS ONE.

[77] Castells M, Schwartz LB (1988). Tryptase levels in nasal-lavage fluid as an indicator of the immediate allergic response. J Allergy Clin Immunol.

[78] Bisgaard H, Grønborg H, Mygind N, Dahl R, Lindqvist N, Venge P (1990). Allergen-induced increase of eosinophil cationic protein in nasal lavage fluid: effect of the glucocorticoid budesonide. J Allergy Clin Immunol.

[79] Bentley AM, Jacobson MR, Cumberworth V, Barkans JR, Moqbel R, Schwartz LB (1992). Immunohistology of the nasal mucosa in seasonal allergic rhinitis: increases in activated eosinophils and epithelial mast cells. J Allergy Clin Immunol.

[80] Gentile DA, Doyle WJ, Fireman P, Skoner DP (2001). Effect of experimental influenza A infection on systemic immune and inflammatory parameters in allergic and nonallergic adult subjects. Ann Allergy Asthma Immunol.

[81] Sigurs N, Bjarnason R, Sigurbergsson F (1994). Eosinophil cationic protein in nasal secretion and in serum and myeloperoxidase in serum in respiratory syncytial virus bronchiolitis: relation to asthma and atopy. Acta Paediatr.

[82] Mygind N, Thomsen J (1976). Diurnal variation of nasal protein concentration. Acta Otolaryngol.

[83] Fairbairn S (2018). Current opinion in allergy and clinical immunology: a change in leadership. Curr Opin Allergy Clin Immunol.

[84] Jochems SP, Piddock K, Rylance J, Adler H, Carniel BF, Collins A (2017). Novel analysis of immune cells from nasal microbiopsy demonstrates reliable, reproducible data for immune populations, and superior cytokine detection compared to nasal wash. PLoS ONE.

[85] Pruski P, MacIntyre DA, Lewis HV (2017). Novel analysis of immune cells from nasal microbiopsy demonstrates reliable, reproducible data for immune populations, and superior cytokine detection compared to nasal wash. PLoS ONE.

[86] Shin YS, Jung CG, Park HS (2018). Prevalence and clinical characteristics of local allergic rhinitis to house dust mites. Curr Opin Allergy Clin Immunol.

[87] König K, Klemens C, Haack M, Nicoló MS, Becker S, Kramer MF (2016). Cytokine patterns in nasal secretion of non-atopic patients distinguish between chronic rhinosinusitis with or without nasal polys. Allergy Asthma Clin Immunol.

[88] Hansel TT, Tunstall T, Trujillo-Torralbo MB, Shamji B, Del-Rosario A, Dhariwal J (2017). A comprehensive evaluation of nasal and bronchial cytokines and chemokines following experimental rhinovirus infection in allergic asthma: increased interferons (IFN-γ and IFN-λ) and type 2 inflammation (IL-5 and IL-13). EBioMedicine.

[89] Succar EF, Turner JH (2018). Recent advances in understanding chronic rhinosinusitis endotypes. F1000Res.

[90] Collier DA, Assennato SM, Warne B, Sithole N, Sharrocks K, Ritchie A (2020). Point of care nucleic acid testing for SARS-CoV-2 in hospitalized patients: a clinical validation trial and implementation study. Cell Rep Med.

[91] Sun F, Ganguli A, Nguyen J, Brisbin R, Shanmugam K, Hirschberg DL (2020). Smartphone-based multiplex 30-minute nucleic acid test of live virus from nasal swab extract. Lab Chip.

[92] Abdulrahman A, Mustafa F, AlAwadhi AI, Alansari Q, AlAlawi B, AlQahtani M (2020). Comparison of SARS-COV-2 nasal antigen test to nasopharyngeal RT-PCR in mildly symptomatic patients. medRxiv.

[93] Young S, Taylor SN, Cammarata CL, Varnado KG, Roger-Dalbert C, Montano A (2020). Clinical evaluation of BD veritor SARS-CoV-2 point-of-care test performance compared to PCR-based testing and versus the Sofia 2 SARS antigen point-of-care test. J Clin Microbiol.

[94] Vadlamani BS, Uppal T, Verma SC, Misra M (2020). Functionalized TiO_2_ nanotube-based electrochemical biosensor for rapid detection of SARS-CoV-2. Sensors.

[95] Yam WC, Siu GK, Ho PL, Ng TK, Que TL, Yip KT (2013). Evaluation of the LightCycler methicillin-resistant *Staphylococcus aureus* (MRSA) advanced test for detection of MRSA nasal colonization. J Clin Microbiol.

[96] Chotiprasitsakul D, Tamma PD, Gadala A, Cosgrove SE (2018). The role of negative methicillin-resistant *Staphylococcus aureus* nasal surveillance swabs in predicting the need for empiric vancomycin therapy in intensive care unit patients. Infect Control Hosp Epidemiol.

[97] Eom G, Hwang A, Kim H, Yang S, Lee DK, Song S (2019). Diagnosis of tamiflu-resistant influenza virus in human nasal fluid and saliva using surface-enhanced Raman scattering. ACS Sens.

[98] Meng Y, Lou H, Wang Y, Wang C, Zhang L (2018). The use of specific immunoglobulin E in nasal secretions for the diagnosis of allergic rhinitis. Laryngoscope.

[99] Hirvonen MR, Ruotsalainen M, Roponen M, Hyvärinen A, Husman T, Kosma VM (1999). Nitric oxide and proinflammatory cytokines in nasal lavage fluid associated with symptoms and exposure to moldy building microbes. Am J Respir Crit Care Med.

[100] Aliste M, Chávez LG (2016). Analysis of gunshot residues as trace in nasal mucus by GFAAS. Forensic Sci Int.

[101] Merli D, Brandone A, Amadasi A, Cattaneo C, Profumo A (2016). The detection of gunshot residues in the nasal mucus of suspected shooters. Int J Legal Med.

[102] D'Elia V, Montalvo G, Ruiz CG (2016). Analysis of street cocaine samples in nasal fluid by Raman spectroscopy. Talanta.

[103] Armenta S, de la Guardia M, Alcalà M, Blanco M (2013). Noninvasive double confirmation of cocaine abuse. Anal Chem.

[104] Hansson KT, Skillbäck T, Pernevik E, Holmén-Larsson J, Brinkmalm G, Blennow K (2017). Sample preparation for endopeptidomic analysis in human cerebrospinal fluid. J Vis Exp.

[105] Vanderstichele H, Demeyer L, Janelidze S, Coart E, Stoops E, Mauroo K (2017). Recommendations for cerebrospinal fluid collection for the analysis by ELISA of neurogranin trunc P75, α-synuclein, and total tau in combination with Aβ(1–42)/Aβ(1–40). Alzheimers Res Ther.

[106] Shahnawaz M, Tokuda T, Waragai M, Mendez N, Ishii R, Trenkwalder C (2017). Development of a biochemical diagnosis of Parkinson disease by detection of α-synuclein misfolded aggregates in cerebrospinal fluid. JAMA Neurol.

[107] Song C, Deng P, Que L (2018). Rapid multiplexed detection of beta-amyloid and total-tau as biomarkers for Alzheimer’s disease in cerebrospinal fluid. Nanomedicine.

[108] Fan S, Ren H, Wei Y, Mao C, Ma Z, Zhang L (2018). Next-generation sequencing of the cerebrospinal fluid in the diagnosis of neurobrucellosis. Int J Infect Dis.

[109] Eichinger A, Hagen A, Meyer-Bühn M, Huebner J (2019). Clinical benefits of introducing real-time multiplex PCR for cerebrospinal fluid as routine diagnostic at a tertiary care pediatric center. Infection.

[110] Ho EL, Tantalo LC, Jones T, Sahi SK, Marra CM (2015). Point-of-care treponemal tests for neurosyphilis diagnosis. Sex Transm Dis.

[111] Rousseau G, Asmolov R, Grammatico-Guillon L, Auvet A, Laribi S, Garot D (2019). Rapid detection of bacterial meningitis using a point-of-care glucometer. Eur J Emerg Med.

[112] Lefrere B, Plantamura J, Renard C, Ceppa F, Delacour H (2017). Biochemical analysis of cerebrospinal fluid in the laboratories of deployed medical treatment facilities: are Multistix 10 SG strip and iSTAT useful?. J R Army Med Corps.

[113] Delacroix R, Morel SN, Hervé L, Bordy T, Dinten JM, Drancourt M (2017). Cerebrospinal fluid lens-free microscopy: a new tool for the laboratory diagnosis of meningitis. Sci Rep.

[114] Turetsky A, Lee K, Song J, Giedt RJ, Kim E, Kovach AE (2015). On chip analysis of CNS lymphoma in cerebrospinal fluid. Theranostics.

[115] Schleh C, Leoni AL (2013). How to optimize the benefits of computer assisted sperm analysis in experimental toxicology. J Occup Med Toxicol.

[116] Su TW, Erlinger A, Tseng D, Ozcan A (2010). Compact and light-weight automated semen analysis platform using lensfree on-chip microscopy. Anal Chem.

[117] Kanakasabapathy MK, Sadasivam M, Singh A, Preston C, Thirumalaraju P, Venkataraman M (2017). An automated smartphone-based diagnostic assay for point-of-care semen analysis. Sci Transl Med.

[118] Karsten SL, Tarhan MC, Kudo LC, Collard D, Fujita H (2015). Point-of-care (POC) devices by means of advanced MEMS. Talanta.

[119] Matsuura K, Huang HW, Chen MC, Chen Y, Cheng CM (2017). Relationship between porcine sperm motility and sperm enzymatic activity using paper-based devices. Sci Rep.

[120] Tsao YT, Yang CY, Wen YC, Chang TC, Matsuura K, Chen Y (2020). Point-of-care semen analysis of patients with infertility via smartphone and colorimetric paper-based diagnostic device. Bioeng Transl Med.

[121] Mitchell C, Paul K, Agnew K, Gaussman R, Coombs RW, Hitti J (2011). Estimating volume of cervicovaginal secretions in cervicovaginal lavage fluid collected for measurement of genital HIV-1 RNA levels in women. J Clin Microbiol.

[122] Anahtar MN, Gootenberg DB, Mitchell CM, Kwon DS (2018). Cervicovaginal microbiota and reproductive health: the virtue of simplicity. Cell Host Microbe.

[123] Amabebe E, Reynolds S, Stern VL, Parker JL, Stafford GP, Paley MN (2016). Identifying metabolite markers for preterm birth in cervicovaginal fluid by magnetic resonance spectroscopy. Metabolomics.

[124] Amabebe E, Reynolds S, Stern V, Stafford G, Paley M, Anumba DO (2016). Cervicovaginal fluid acetate: a metabolite marker of preterm birth in symptomatic pregnant women. Front Med.

[125] Yoo HN, Park KH, Jung EY, Kim YM, Kook SY, Jeon SJ (2017). Non-invasive prediction of preterm birth in women with cervical insufficiency or an asymptomatic short cervix (≤25 mm) by measurement of biomarkers in the cervicovaginal fluid. PLoS ONE.

[126] Amabebe E, Chapman DR, Stern VL, Stafford G, Anumba DOC (2018). Mid-gestational changes in cervicovaginal fluid cytokine levels in asymptomatic pregnant women are predictive markers of inflammation-associated spontaneous preterm birth. J Reprod Immunol.

[127] Ryu A, Park KH, Oh KJ, Lee SY, Jeong EH, Park JW (2013). Predictive value of combined cervicovaginal cytokines and gestational age at sampling for intra-amniotic infection in preterm premature rupture of membranes. Acta Obstet Gynecol Scand.

[128] Choi SR, Hong SS, Kim J, Lee KY (2018). Neutrophil elastase in cervical fluid in women with short cervical length. Taiwan J Obstet Gynecol.

[129] Kacerovsky M, Musilova I, Jacobsson B, Drahosova M, Hornychova H, Janku P (2015). Vaginal fluid IL-6 and IL-8 levels in pregnancies complicated by preterm prelabor membrane ruptures. J Matern Fetal Neonatal Med.

[130] Kacerovsky M, Musilova I, Jacobsson B, Drahosova M, Hornychova H, Janku P (2015). Cervical fluid IL-6 and IL-8 levels in pregnancies complicated by preterm prelabor rupture of membranes. J Matern Fetal Neonatal Med.

[131] Combs CA, Garite TJ, Lapidus JA, Lapointe JP, Gravett M, Rael J (2015). Detection of microbial invasion of the amniotic cavity by analysis of cervicovaginal proteins in women with preterm labor and intact membranes. Am J Obstet Gynecol.

[132] Jung EY, Park JW, Ryu A, Lee SY, Cho SH, Park KH (2016). Prediction of impending preterm delivery based on sonographic cervical length and different cytokine levels in cervicovaginal fluid in preterm labor. J Obstet Gynaecol Res.

[133] Lee SM, Park KH, Jung EY, Kook SY, Park H, Jeon SJ (2018). Inflammatory proteins in maternal plasma, cervicovaginal and amniotic fluids as predictors of intra-amniotic infection in preterm premature rupture of membranes. PLoS ONE.

[134] Musilova I, Bestvina T, Hudeckova M, Michalec I, Cobo T, Jacobsson B (2016). Vaginal fluid IL-6 concentrations as a point-of-care test is of value in women with preterm PROM. Am J Obstet Gynecol.

[135] Moncla BJ, Chappell CA, Mahal LK, Debo BM, Meyn LA, Hillier SL (2015). Impact of bacterial vaginosis, as assessed by nugent criteria and hormonal status on glycosidases and lectin binding in cervicovaginal lavage samples. PLoS ONE.

[136] Tyssen D, Wang YY, Hayward JA, Agius PA, DeLong K, Aldunate M (2018). Anti-HIV-1 activity of lactic acid in human cervicovaginal fluid. mSphere.

[137] Łaniewski P, Cui H, Roe DJ, Barnes D, Goulder A, Monk BJ (2019). Features of the cervicovaginal microenvironment drive cancer biomarker signatures in patients across cervical carcinogenesis. Sci Rep.

[138] Appidi T, Mudigunda SV, Kodandapani S, Rengan AK (2020). Development of label-free gold nanoparticle based rapid colorimetric assay for clinical/point-of-care screening of cervical cancer. Nanoscale Adv.

[139] Chang H, Zheng M, Yu X, Than A, Seeni RZ, Kang R, Tian J, Khanh DP, Liu L, Chen P, Xu C (2017). A swellable microneedle patch to rapidly extract skin interstitial fluid for timely metabolic analysis. Adv Mater.

[140] Yetisen AK, Moreddu R, Seifi S, Jiang N, Vega K, Dong X, Dong J, Butt H, Jakobi M, Elsner M, Koch AW (2019). Dermal tattoo biosensors for colorimetric metabolite detection. Angew Chem Int Ed Engl.

[141] Kolluru C, Williams M, Yeh JS, Noel RK, Knaack J, Prausnitz MR (2019). Monitoring drug pharmacokinetics and immunologic biomarkers in dermal interstitial fluid using a microneedle patch. Biomed Microdevices.

